# The Interactive Effects of Crude Oil and Corexit 9500 on Their Biodegradation in Arctic Seawater

**DOI:** 10.1128/AEM.01194-20

**Published:** 2020-10-15

**Authors:** Taylor R. Gofstein, Matthew Perkins, Jennifer Field, Mary Beth Leigh

**Affiliations:** a Institute of Arctic Biology, University of Alaska Fairbanks, Fairbanks, Alaska, USA; b Department of Chemistry & Biochemistry, University of Alaska Fairbanks, Fairbanks, Alaska, USA; c Department of Environmental and Molecular Toxicology, Oregon State University, Corvallis, Oregon, USA; University of California, Davis

**Keywords:** oil, Corexit 9500, biodegradation, Arctic, chemical dispersants, crude oil

## Abstract

The risk of petroleum spills coupled with the potential application of chemical dispersants as a spill response strategy necessitates further understanding of the fate of oil and dispersants and their interactive effects during biodegradation. Using Arctic seawater mesocosms amended with either crude oil, Corexit 9500, or both together, we quantified the chemical losses of crude oil and Corexit 9500 and identified microbial taxa implicated in their biodegradation based on shifts in the microbial community structure over a 30-day time course. Chemical analyses included total petroleum hydrocarbons (TPH), *n*-alkanes, branched alkanes, and polycyclic aromatic hydrocarbons (PAHs) for oil loss and the surfactant components dioctyl sodium sulfosuccinate (DOSS), Span 80, Tween 80, Tween 85, and the DOSS metabolite ethylhexyl sulfosuccinate (EHSS) for Corexit loss. Changes to the microbial communities and identification of key taxa were determined by 16S rRNA gene amplicon sequencing. The nonionic surfactants of Corexit 9500 (Span 80 and Tweens 80 and 85) biodegraded rapidly, dropping to below the limits of detection within 5 days and prior to any detectable initiation of oil biodegradation. This resulted in no observable suppression of petroleum biodegradation in the presence of Corexit compared to that of oil alone. In contrast, biodegradation of DOSS was delayed in the presence of oil, based on the prolonged presence of DOSS and accumulation of the degradation intermediate EHSS that did not occur in the absence of oil. Microbial analyses revealed that oil and Corexit enriched different overall microbial communities, with the presence of both resulting in a community composition that shifted from one more similar to that of Corexit only to one reflecting the oil-only community over time, in parallel with the degradation of predominantly Corexit and then oil components. Some microbial taxa (*Oleispira, Pseudofulvibacter*, and *Roseobacter*) responded to either oil or Corexit, suggesting that some organisms may be capable of utilizing both substrates. Together, these findings reveal interactive effects of crude oil and Corexit 9500 on chemical losses and microbial communities as they biodegrade, providing further insight into their fate when copresent in the environment.

**IMPORTANCE** Chemical dispersants such as Corexit 9500 are commonly used in oil spill response and are currently under consideration for use in the Arctic, where their fate and effects have not been well studied. This research was performed to determine the interactive effects of the copresence of crude oil and Corexit 9500 on the degradation of components from each mixture and the associated microbial community structure over time in Arctic seawater. These findings will help yield a better understanding of the biodegradability of dispersant components applied to an oil spill, the temporal microbial community response to dispersed oil, and the fundamental microbial ecology of organic contaminant biodegradation processes in the Arctic marine environment.

## Abstract (precis)

Chemical dispersants such as Corexit 9500 are commonly used in oil spill response and are currently under consideration for use in the Arctic, where their fate and effects have not been well studied. This research was performed to determine the interactive effects of the copresence of crude oil and Corexit 9500 on the degradation of components from each mixture and the associated microbial community structure over time in Arctic seawater. These findings will help yield a better understanding of the biodegradability of dispersant components applied to an oil spill, the temporal microbial community response to dispersed oil, and the fundamental microbial ecology of organic contaminant biodegradation processes in the Arctic marine environment.

## INTRODUCTION

The chemical dispersant Corexit EC9500A (here referred to as Corexit 9500 or Corexit) has garnered increased interest in recent years as an oil spill response strategy and is currently under consideration for use in Alaskan Arctic waters. Chemical dispersants have utility in preventing surface oil slicks from reaching shorelines as well as the potential to enhance oil biodegradation by increasing oil bioavailability (1). Yet there remain concerns regarding possible unintended ecological consequences from their usage, particularly when the fate and effects of dispersants in the environment are not well characterized.

To date, several studies have reported increased rates of petroleum biodegradation with the addition of chemical dispersants, including in the Arctic (2, 3), using either enrichment cultures (4–7) or indigenous microbial communities (1–3, 8–11), the latter of which are more likely to better reflect rates observed for *in situ* conditions. Recently, some studies appear to demonstrate the opposite effect, with dispersant addition negatively affecting oil biodegradation in indigenous (12) and cultured (13) seawater through processes such as suppressing the growth of some oil-degrading bacteria or competitive substrate biodegradation, reintroducing some debate regarding the influence of chemical dispersants on petroleum biodegradation.

While much is known about oil biodegradation (14–17), considerably less is known about the biodegradation of Corexit 9500. This dispersant formulation is composed of the anionic surfactant dioctyl sodium sulfosuccinate (DOSS; 18% [wt/wt]) and the nonionic surfactants Span 80 (4.4% [wt/wt]), Tween 80 (18% [wt/wt]), and Tween 85 (4.6% [wt/wt]) in a petroleum distillate solvent (18, 19). Additionally, ethylhexyl sulfosuccinate (EHSS) has been identified as a degradation intermediate of DOSS (4, 20) and is also present in detectable quantities (0.28% [wt/wt]) in Corexit 9500 formulations (18). Several studies have measured Corexit component losses in mesocosm experiments over time but have either been limited to only DOSS (4, 7), used enrichment cultures (4), or lacked abiotic controls to determine whether the losses observed are attributable to biodegradation (2, 7, 12). Recently, a study in our lab using biotic and abiotic mesocosms of seawater exposed to Corexit alone suggested that biodegradation of all four surfactant components can occur in the Arctic but did not evaluate the biodegradability and potential interactive effects when oil and Corexit are copresent (3).

Like biodegradation rates and extents, the microbial community shifts and organisms associated with Corexit biodegradation are much less extensively characterized than those that degrade oil. Over 320 bacterial genera across 7 phyla have been identified as capable of degrading petroleum hydrocarbons to date (21). Following the Deepwater Horizon spill and subsequent Corexit 9500 and 9527 application in the Gulf of Mexico, distinct shifts of gammaproteobacteria were observed *in situ*, which were initially dominated by an *Oceanospirillaceae* genus (later identified as *Oleispira*) (22) and *Pseudomonas*, followed by *Colwellia*, *Cycloclasticus*, and *Pseudoalteromonas*; however, it is unknown which of these genera were stimulated by the presence of Corexit versus oil (23). Subsequent incubation experiments have identified several taxa that are stimulated by the presence of Corexit, including *Marinobacter* (24), *Pseudoalteromonas* (24), and *Winogradskyella* (7) in temperate regions, *Oleispira* (3), *Polaribacter* (3), and *Moritella* (3) in the Arctic, and *Colwellia* (3, 7, 12) in both. Many of these genera contain members which are known petroleum hydrocarbon degraders (21), suggesting that some organisms may be capable of degrading both oil and Corexit. Yet, a distinct separation of community structure as a whole has been observed between incubations containing oil or Corexit separately (3, 7, 12, 24) as well as oil with or without dispersant (7, 11, 12, 24), suggesting that the presence of oil, Corexit, and the combination of both may each stimulate unique microbial communities implicated in their degradation.

It is not yet known how the presence of oil and Corexit 9500 together may affect the biodegradation of each in Arctic waters, for example, through processes such as preferential degradation and/or enrichment or suppression of microbes that degrade the other mixture, ultimately affecting the fate of these chemicals in the environment. In this study, we investigate the interactive effects of Alaska North Slope crude oil and Corexit 9500 in Arctic seawater mesocosm incubations on chemical losses and microbial communities, including examination of sequential and codegradation of individual components of oil and Corexit and the identification of microbial taxa putatively involved in the biodegradation of oil and dispersant.

## RESULTS AND DISCUSSION

### Biodegradation of crude oil.

**FIG 1:**
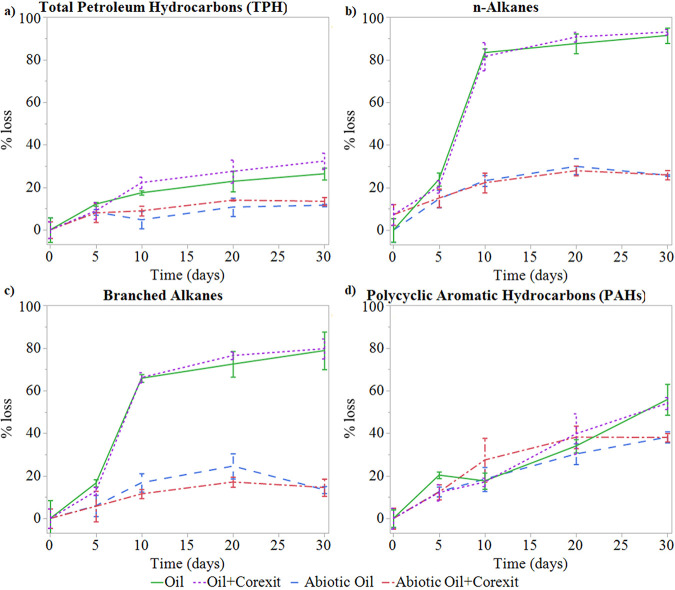
Mean (*n* = 3) percent loss of total petroleum hydrocarbons (TPH) (a), *n*-alkanes (b), branched alkanes (c), and polycyclic aromatic hydrocarbons (PAHs) (d). The error bars denote one standard deviation from the mean.

Incubation experiment mesocosms consisted of 800 ml of Chukchi seawater amended with 16 ppm Bushnell-Haas medium and either 50 ppm Alaska North Slope crude oil, 5 ppm Corexit 9500, both (1:20 dispersant-to-oil ratio), or no substrates, along with abiotic controls of autoclaved seawater, which were destructively harvested at 0, 5, 10, 20, and 30 days. Biodegradation of crude oil and the influence of Corexit 9500 on oil degradation rates were assessed by quantifying the total petroleum hydrocarbon (TPH) (C_10_ to C_30_), *n*-alkane, branched alkane, and polycyclic aromatic hydrocarbon (PAH) losses over time (Fig. 1). While the majority of the alkanes biodegraded within the first 10 days, the remaining TPH and PAHs degraded much more slowly over the course of the 30-day incubation. Evidence of biodegradation contributing to TPH loss was detectable on day 10, which was the first time point in which live treatments experienced greater loss (20% ± 3%) than abiotic treatments (7% ± 4%) (*P* = 0.0002) (Fig. 1a). By the end of the experiment, biotic TPH loss had reached 29% ± 4% and was significantly higher (*P* < 0.0001) than loss in abiotic treatments (12% ± 2%). Based on these findings, both biotic and abiotic processes play important roles in the early losses of crude oil in the Arctic, with biodegradation becoming significant between 5 and 10 days for TPH.

The loss of individual petroleum compounds followed the well-documented pattern of degradation, with smaller, simpler, and saturated hydrocarbon compounds degrading more readily than larger, complex, and conjugated compounds, such that loss of *n*-alkanes was greater than loss of branched alkanes, which was greater than loss of PAHs (see Table S2 in the supplemental material). The rapid degradation of *n*-alkanes but little change in PAH concentrations has also been observed in other Arctic studies under a variety of experimental conditions (25–27). When comparing the loss of the different hydrocarbon compound classes to TPH loss, the remaining hydrocarbons present in live treatments at 30 days appear to be largely composed of PAHs and other recalcitrant hydrocarbons present in the unresolved complex mixture. While linear and branched alkanes show relatively rapid degradation by Arctic microorganisms, the slower degradation of the remaining oil components raises concern for the long-term effects in the environment due to the toxic and bioavailable nature of these compounds (28–31).

These findings support previous studies reporting that marine crude oil biodegradation can occur in the Arctic but more slowly than in temperate regions. In a similarly designed experiment by McFarlin et al. (2), 2.5 ppm oil in −1°C Alaskan Arctic seawater from the same location as this study saw TPH percent losses of 36% and 45% after 10 and 28 days, respectively. We observed slightly lower extents of oil degradation than McFarlin et al., at 20% ± 3% after 10 days and 29% ± 4% after 30 days. While both studies were performed using Arctic seawater at similar temperatures, the lower percentages of TPH loss observed here may be attributable to the higher concentrations of oil used (50 ppm in this study versus 2.5 ppm by McFarlin et al. [2]) and potentially to increased weathering of our stock Alaska North Slope (ANS) crude oil supply over time. Studies from other cold high-latitude regions have observed a similar range of TPH loss in incubation experiments, with a 32% TPH loss by 71 days in Disko Bay (Greenland) (27), an 18% total extractable organic carbon (TEOC) loss by 64 days in the Van Meijen fjord (Svalbard, Norway) (32), and a 39% total extractable material (TEM) loss after 64 days in Trondheimsfjord (Trondheim, Norway) (33). Although they were performed under a variety of experimental conditions, all of these Arctic studies show lower extents of oil loss than in temperate regions. For example, a similarly designed incubation of New Jersey seawater with 2.5 ppm of ANS crude oil at 8°C observed 51% and 69% TPH loss at 11 and 24 days, respectively (8).

While no differences in TPH, *n*-alkane, branched alkane, or PAH loss were observed with the addition of Corexit in this experiment, several similarly designed seawater incubation studies have noted some dispersant-associated effects. McFarlin et al. (2) observed increased TPH loss with the addition of Corexit at 10 days (47% with Corexit, 36% without) and 28 days (54% with Corexit, 45% without). Similarly, Prince et al. (8) also observed an increased TPH loss at 11 (64% with Corexit, 51% without) and 24 (77% with Corexit, 69% without) days with the addition of Corexit. In both of these studies, lower oil concentrations than described here were used and oil and Corexit were premixed prior to their addition to seawater, while in the present study, oil and Corexit were added separately to incubations, which may have an influence on the effects of Corexit on oil dispersion and degradation. Recently, some studies have observed inhibition of oil degradation with the addition of Corexit (12, 13). However, the experimental conditions used here differ substantially from those in which inhibition was reported (e.g., sealed bottles [12, 13], higher oil concentrations [12, 13], the use of water-accommodated fractions rather than whole oil [12], and enrichment cultures [13]), precluding a direct comparison with this study. However, it is important to note that the incubation test methods applied in our studies are not optimized to replicate a comparison of a slick to dispersed oil (1) but rather to mimic the relatively low concentrations of oil and dispersant associated with a dispersed oil plume in order to provide estimates of whole oil biodegradation following dispersion and to assess the sequence and potential interactions of petroleum and dispersant component biodegradation in that context.

### Biodegradation of Corexit 9500.

**FIG 2:**
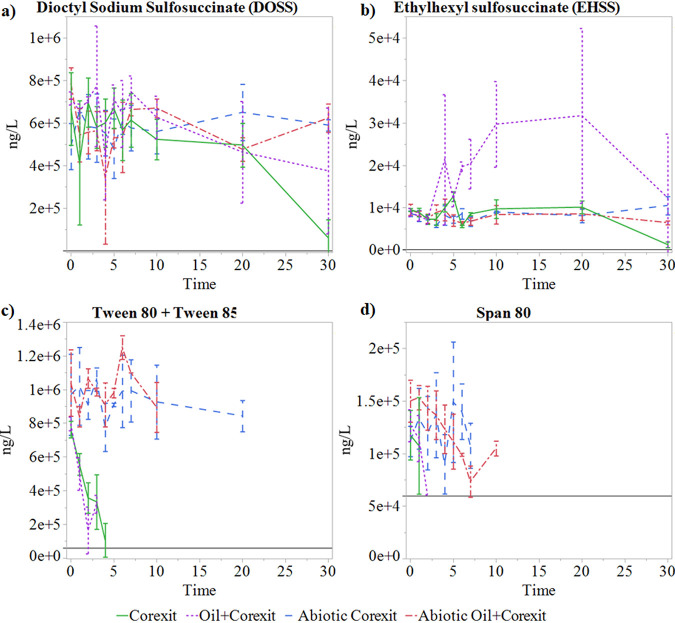
Mean (*n* = 3) concentrations in subsampled 6-liter mesocosms over time of Corexit 9500 constituents or suspected metabolites dioctyl sodium sulfosuccinate (DOSS) (a), ethylhexyl sulfosuccinate (EHSS) (b), Tweens 80 and 85 (c), and Span 80 (d). The error bars denote one standard deviation from the mean, and the horizontal lines in each panel represent the limit of detection (LOD) for that analyte (200 ng/liter for DOSS and EHSS, 60,000 ng/liter for the Tweens and Span 80). All analyses were performed throughout the duration of the experiment, even after concentrations were found to be <LOD.

The biodegradation of Corexit 9500 components was studied with both whole-bottle harvested mesocosms (0, 5, and 30 days) as well as large 6-liter subsampled mesocosms (0, 1, 2, 3, 4, 5, 6, 7, 10, 20, and 30 days). Liquid chromatography-tandem mass spectrometry (LC-MS/MS) was used to quantitate the Corexit surfactant components dioctyl sodium sulfosuccinate (DOSS), Span 80, Tween 80, and Tween 85 as well as the DOSS metabolite ethylhexyl sulfosuccinate (EHSS). Analyses of the surfactant constituents of Corexit 9500 revealed rapid degradation of the nonionic surfactants but were inconclusive for DOSS due to high analytical variability between replicates (Fig. 2). Concentrations of DOSS in individual samples were consistent with the previously reported accuracy for the analytical methods (18) yet were also variable in the whole-bottle mesocosms as well, suggesting that DOSS measurement variation may occur as an innate property of the compound, such as its surface-active behavior, resulting in significant sorption to mesocosm walls.

The nonionic surfactants in Corexit (Tween 80, Tween 85, and Span 80) showed rapid losses with and without the presence of oil. Tween 80 and 85 concentrations (Fig. 2c) had fallen below the limits of detection (LOD) in both biotic treatments by day 5 (60,000 ng/liter), and Span 80 (Fig. 2d) dropped below the LOD (60,000 ng/liter) by day 3, suggesting that rapid degradation of both components can occur in Arctic marine environments. Decreases of all nonionic surfactants in abiotic treatments by 30 days was also observed, suggesting that there is abiotic transformation and loss of these analytes occurring in addition to biotic processes.

Based on initial concentrations, measurements below the LOD represented a ≥93% loss for the Tweens at 5 days in biotic treatments and 30 days for abiotic treatments. However, the extent of the loss of Span 80 below the LOD is more uncertain, with the LOD representing ≥53% loss based on initial concentrations. This LOD was reached at 3 days in biotic treatments and 20 days in abiotic treatments. The rapid loss of the nonionic surfactants demonstrated here has also been observed in previous seawater incubation experiments. Kleindienst et al. (12) observed concentrations of Tweens and Span 80 falling below detection limits (20 μg/liter for Tweens and 36 μg/liter Span 80) within 1 week for Gulf of Mexico seawater at 8°C; based on the starting concentrations of these compounds, this represents an ∼99.7% loss of the Tweens and ∼87% loss of Span 80. McFarlin et al. (3) also saw near complete (>97%) loss of Span 80 in 28 days at 2°C for both nearshore and offshore Arctic seawater. The loss of Span 80 was largely attributable to biodegradation due to much lower losses observed for abiotic treatments (46% offshore and 1% nearshore at 28 days) (3). The Tweens also underwent rapid loss, although less of this is attributable to biodegradation, with >99% total loss and 82% abiotic loss observed for nearshore seawater at 10 days (3). Additionally, Brakstad et al. observed rapid Tween loss (>96% at 4 days) in Trondheimsfjord seawater at 5°C but also a 16-day lag period in DOSS degradation (34). DOSS loss was concentration dependent, with 16% loss at 1 ppm and 4% loss at 50 ppm over 54 days (34). DOSS and Tween loss were both attributable to biodegradation due to little change in abiotic treatments over time (34). These findings suggest that the nonionic surfactant components of Corexit are rapidly lost from the environment through both biotic and abiotic processes, although the pathways and mechanisms underlying this have yet to be determined.

In both destructively harvested and subsampled incubations, DOSS concentrations showed no significant differences between treatments over time until 30 days (Fig. 2a). At the end of the 30-day incubation, a 91% ± 13% loss of DOSS was observed for Corexit alone, and 48% ± 41% loss was observed when Corexit was incubated in the presence of oil. The loss of DOSS in Corexit-only treatments was significantly higher (*P* = 0.0018) than in the abiotic controls (0% ± 23% loss), suggesting biodegradation as a mechanism for loss. Biotic losses of DOSS did not exceed abiotic losses when Corexit was in the presence of oil, but it was also noted during analysis that recovery and measurement of DOSS in treatments containing oil were more challenging than with Corexit alone, contributing to the variation observed here. However, EHSS, a metabolite of DOSS, accumulated to significantly higher levels in treatments containing both crude oil and Corexit than in Corexit-only treatments by day 6 (*P* < 0.0001) in subsampled incubations and remained high throughout the remainder of the experiment in both subsampled and destructively harvested incubation types (Fig. 2b). This increase in EHSS concentrations suggests that, although direct quantitation of DOSS itself was inconclusive due to variation in the data, DOSS transformation may in fact be occurring. Interestingly, EHSS concentrations increased for treatments containing both crude oil and Corexit but did not increase for treatments containing Corexit only. The prolonged presence of EHSS and decreased DOSS loss suggests that further DOSS mineralization beyond EHSS was delayed or incomplete in the presence of oil.

As in our study, large variations for DOSS measurements between replicates have been observed by others (4, 12), underscoring the difficulty of reliably quantifying it in experiments. The extent of DOSS degradation has also varied considerably depending on the experimental conditions. For example, McFarlin et al. (3) performed Arctic seawater incubations at 2°C and observed 98% total loss and 21% abiotic loss of DOSS in offshore seawater at 28 days, yet only observed 35% total and 2% abiotic loss in nearshore seawater. In experiments using Gulf of Mexico seawater at 8°C, Kleindienst et al. (12) found an 8% loss of DOSS for Corexit alone and ∼30% loss of DOSS for Corexit with oil at 4 weeks. In an experiment using cultures isolated near the Macondo wellhead in the Gulf of Mexico by Campo et al. (4), DOSS did not undergo substantial degradation within 28 days at 5°C; however, at 25°C, the cultures exhibited rapid and complete DOSS degradation within 14 days. Similar results were observed by Techtmann et al. (7), who used the same cultures and experimental conditions, with the majority of degradation at 25°C occurring within 20 days, but no observable degradation at 5°C over the course of the 56-day experiment. Our current and previous studies (3) demonstrated little abiotic loss of DOSS in Arctic seawater. This is further supported by evidence that DOSS does not undergo significant hydrolysis or photolytic degradation under simulated solar conditions (35, 36), suggesting that biodegradation largely determines the fate of DOSS released into the environment.

While some mesocosm- and culture-based experiments have revealed rapid degradation of DOSS, *in situ* measurements in the Gulf of Mexico following the Deepwater Horizon oil spill suggest that DOSS may be more recalcitrant in the environment. Water samples collected by Kujawinski et al. (37) 64 days after dispersant applications had ceased for the Deepwater Horizon spill demonstrated the persistence of DOSS, which was present in concentrations orders of magnitude higher than those that were predicted by dilution and transport. Additionally, White et al. (38) observed DOSS concentrations ranging from 6 to 9,000 ng/g in coral communities 6 months after the spill and 1 to 260 ng/g in beach sands 26 to 45 months after. The presence of DOSS in Gulf of Mexico sediments was confirmed by Perkins et al. (39) along with the discovery of DOSS in settling particles (39). This disparity between the results of laboratory experiments and *in situ* measurements may be due to laboratory conditions being unable to accurately replicate environmental conditions. Based on the findings to date, it remains unclear if DOSS degrades, whether biotically or abiotically, to an appreciable extent in the environment when large quantities are applied following an oil spill.

During TPH analysis, an unexpected set of peaks was detected by gas chromatography (GC)-MS in treatments of oil with the addition of Corexit that were not present in oil-only treatments. The compound represented by these peaks was identified based on its mass spectrum as 1-(2-butoxy-1-methylethoxy)propan-2-ol (see Fig. S1), also known as dipropylene glycol *n*-butyl ether (DGBE). DGBE is an industrial chemical used as a solvent, chemical reaction intermediate, insecticide, and surfactant and has been identified as a solvent component of Corexit 9500 (14). This compound remained in significant abundance at the end of the incubation series relative to the components of the crude oil (Fig. S1). Owing to its more complex chemical structure, DGBE may be resistant to biodegradation in the presence of more labile compounds in crude oil and Corexit such as *n*-alkanes and nonionic surfactants. Loss of this compound cannot be accurately quantitated from the analytical methods used here, which are intended to measure the loss of oil compounds normalized to an internal biomarker present only within crude oil and the variability of concentrations in individual mesocosms due to the extremely small volumes of Corexit used. However, it is still noteworthy that DGBE is detectable in significant amounts relative to crude oil compounds by the end of the 30-day incubation, which is an important environmental consideration. While there have not been any mesocosm-based experiments performed to study DGBE degradation, *in situ* persistence has also been observed in nearshore water and sediment samples and offshore water samples 4 months after the Deepwater Horizon spill (40). Although DGBE has a relatively low acute toxicity compared to that of oil, it has documented acute effects on the liver, and the effects of long-term chronic exposure are unknown (41, 42). This component of Corexit has not been studied as extensively as other constituents and represents a gap in the literature that future work should fill in order to better understand its fate.

Based on the findings of these chemical analyses, there appears to be significant interactive effects on substrate biodegradation when oil and Corexit are copresent. While oil biodegradation was not depressed by the presence of Corexit, the nonionic surfactant components of Corexit were degraded prior to significant oil degradation. The degradation of the nonionic surfactants Span 80 and Tweens 80 and 85 do not appear to be affected by the presence of oil. However, the presence of oil appears to significantly affect the degradation of DOSS, resulting in the temporary accumulation of a degradation intermediate and a lower percent loss of DOSS than in the absence of oil. Following application of Corexit 9500 to an oil spill, the sequence of chemical biodegradation likely begins with Tween 80, Tween 85, and Span 80 before proceeding to the degradation of labile oil compounds such as *n*-alkanes and branched alkanes and continues on with the degradation of more recalcitrant components such as PAHs in oil and DOSS and DGBE in Corexit.

### Bacterial and archaeal microbial community structure.

**FIG 3:**
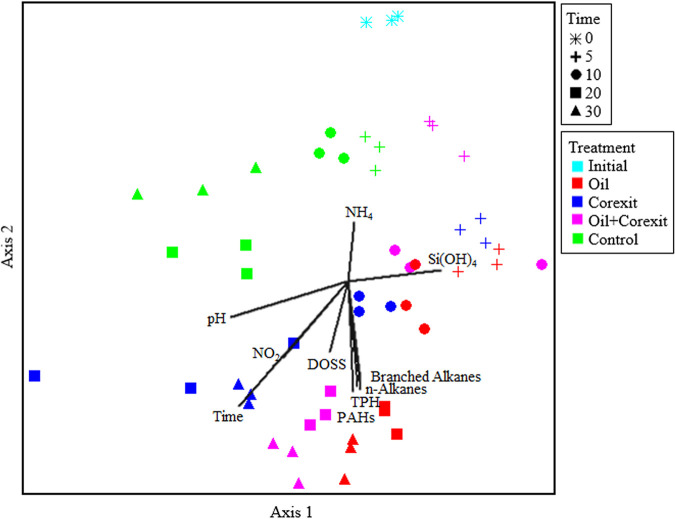
Bray-Curtis NMDS ordination of the prokaryotic microbial community structure and correlating environmental parameters.

**FIG 4:**
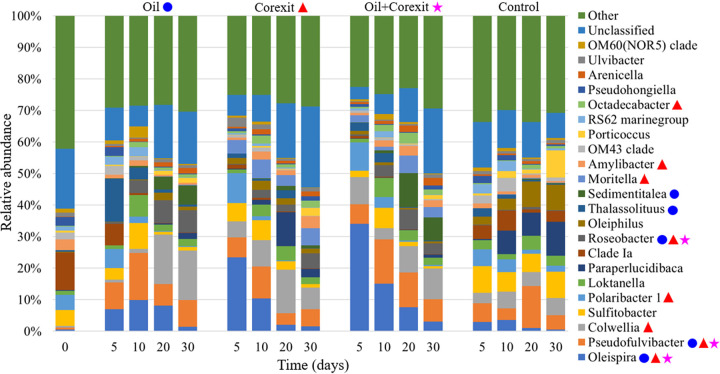
Mean (*n* = 3) relative abundance of prokaryotic microbial community taxa at genus level for experimental treatments over time. Genera which were present in significantly higher (*P* < 0.05) abundances in a particular treatment are denoted with colored symbols as proliferating in the presence of oil alone (blue circles), Corexit 9500 alone (red triangles), or both together (magenta stars).

**FIG 5:**
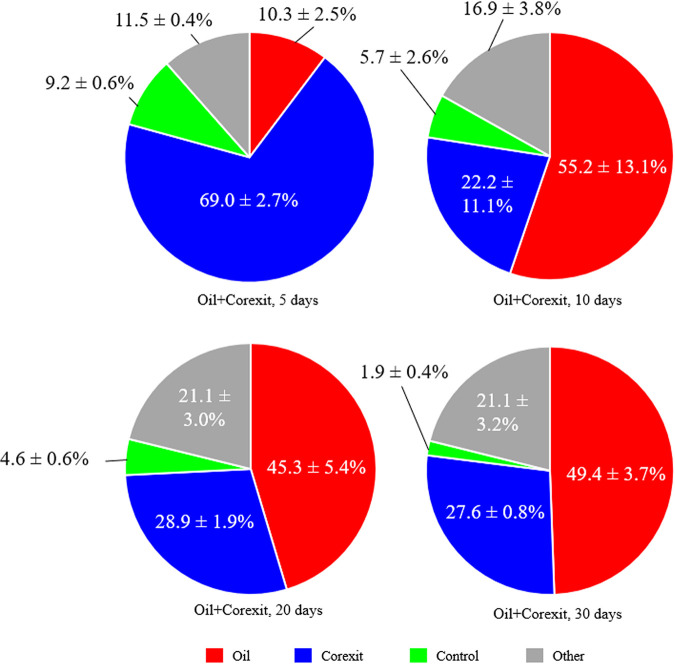
Average (*n* = 3) proportions of the microbial community structure attributable to the presence of oil or Corexit 9500 in Arctic seawater exposed to both over time. Source proportions were determined by comparing oil+Corexit samples against oil-only, Corexit-only, and control samples using the SourceTracker R Bayesian source tracking statistical package.

Changes in the prokaryotic microbial community structure were evaluated by analyzing bacterial and archaeal 16S rRNA gene amplicon sequences, which revealed significant effects of the experimental treatments applied as well as incubation time on microbial community succession (Fig. 3). Of note are the communities associated with the copresence of oil and Corexit (here referred to as oil+Corexit) treatment, which were similar in structure to those with Corexit-only treatments early on, and then shifted toward a community similar to that with oil-only treatments as the incubation progressed (Fig. 3 to 5). Generally, different taxa responded to the presence of either oil or Corexit, although some taxa and amplicon sequence variants (ASVs) responded to both (Fig. 4; see also Table S4). In addition to Corexit and oil, several other environmental parameters also correlated with community structure and the proliferation of specific taxa, including nitrogen compounds, dissolved oxygen, and pH (Fig. 3; see also Table S5).

When comparing the community structures of all samples, they appear to cluster by treatment (Fig. 3), where the oil+Corexit communities are grouped between the oil-only and Corexit-only communities in ordination space. A multiresponse permutation procedure (MRPP) test demonstrated the observed clustering was statistically significant (*P* < 0.0001) and all pairwise comparisons were significant (*P* < 0.032), indicating that the microbial communities of each treatment were significantly different from one another. A permutational multivariate analysis of variance (PERMANOVA) test of all treatments and time points found significant effects of treatment (*P* = 0.0002), time (*P* = 0.0002), and the interactions of both (*P* = 0.0002), indicating that in addition to the treatments applied, time is also a significant factor driving the microbial community structure, indicative of succession. To isolate the influence of the treatments applied, comparisons were also made at each time point. MRPPs showed a significant difference between treatments (*P* < 0.0004 for each time point), and all pairwise comparisons were significant (*P* < 0.033) except for oil and oil+Corexit at day 10 (*P* = 0.8920).

The communities associated with the oil+Corexit treatment contained a combination of the genera stimulated by the presence of oil or Corexit alone (Fig. 4 and 5). The SourceTracker Bayesian statistical source tracking R package (43) was used to determine the proportions of the oil+Corexit community structure attributable to the presence of oil only versus Corexit only. At day 5, the oil+Corexit community was more similar to that of the Corexit-only treatments, with 69.0% ± 2.7% of the community makeup attributable to exposure to Corexit and only 10.3% ± 2.5% of the community attributable to exposure to oil. However, after 10 days, the oil+Corexit community became more similar to that of the oil-only treatments, with 55.2% ± 13.1% of the community attributable to the presence of oil and only 22.2% ± 11.1% of the community makeup attributable to the presence of Corexit (Fig. 5). The chemical analyses mirror this, showing a rapid loss of the nonionic surfactants in Corexit due to degradation within the first 5 days and oil not undergoing extensive degradation until after 10 days. This pattern of community changes and chemical losses suggests that these labile Corexit components are initially degraded preferentially over the less-labile oil compounds (12); however, this did not appear to negatively impact oil degradation itself, at least not at the resolution of our analysis. Previous studies have also found a distinct separation of community structures between incubations containing oil or Corexit separately (3, 7, 12) and those containing oil and Corexit, representing an overlap between the two groups (7).

There were several notable taxa that significantly increased (*P* < 0.05) in relative abundance in response to Corexit compared to that in controls, including *Colwellia*, *Polaribacter*, *Moritella*, *Octadecabacter*, and *Amylibacter* (Fig. 4). Taxa that experienced significant (*P* < 0.05) increases in response to oil included *Thalassolituus* and *Sedimentitalea*. There were also some taxa which significantly increased (*P* < 0.05) in abundance in the presence of oil, Corexit, or both combined, including *Oleispira*, *Pseudofulvibacter*, and *Roseobacter*. Additionally, backward stepwise model selection for individual genera revealed that the relative abundances of organisms stimulated by the presence of oil or Corexit were predicted by concentrations of those respective chemical components as well as nutrients (see Table S6), and genera stimulated by both oil and Corexit were found to be predicted by compounds from both, implicating these taxa as potential biodegradative organisms. For these shared taxa, individual ASVs were frequently shared across all experimental treatments and enriched relative to that in controls (see Table S4). ASVs that were identified to be unique to either oil or Corexit treatments by indicator species analysis were found to be present in treatments containing both (see Tables S7 to S9), indicating that the addition of Corexit did not suppress the proliferation of oil-degrading bacteria.

Many of the taxa stimulated by oil or Corexit here have been observed in previous *in situ* measurements and incubation studies from a variety of marine environments, including *Oleispira* (3, 22, 32, 44, 45), *Colwellia* (3, 12, 22–24, 46, 47), *Moritella* (3), *Octadecabacter* (46), *Thalassolituus* (24, 44, 45, 48, 49), and *Roseobacter* (45, 49) as well as numerous others not observed here such as *Cycloclasticus* (12, 23, 24, 32, 45, 47–49), *Pseudomonas* (23, 50–52), and *Marinobacter* (7, 12, 24, 48, 49, 51, 52). The response of *Amylibacter* to Corexit and *Pseudofulvibacter* (previously unclassified) to both oil and Corexit described here has not been previously reported. While some taxa have been demonstrated thus far to only be stimulated by oil (i.e., *Thalassolituus* [45, 49] and *Cycloclasticus* [10, 12, 24, 45, 49]) or Corexit (i.e., *Moritella* [3]), others have been found to be stimulated by the presence of either or both together (i.e., *Colwellia* [3, 12, 22, 24, 46], *Oleispira* [3, 22, 32, 45], and *Polaribacter* [3, 10, 53]) in different experiments. The variety of different responding taxa observed may be attributable to biogeographical differences as well as the multitude of environmental conditions that can influence community succession. For example, in samples originating from the Gulf of Mexico, Redmond and Valentine (47) observed that the abundance of *Colwellia* was inversely proportional to temperature, and Techtmann et al. (7) also observed a dramatic influence of temperature on community composition. In a comparison of Arctic, Gulf of Mexico, and tropical seawaters, Sun and Kostka observed significant effects of sample site, temperature, and nutrient availability on microbial community composition, with *Colwellia* particularly prominent in Arctic waters at expected ambient conditions (4 to 8°C) (54). Yakimov et al. (55) has also noted that biogeography influences communities, with *Cycloclasticus* and *Thalassolituus* being widely distributed but *Oleispira* more common at high latitudes. The sharing of phylogenetic units (oligotypes, operational taxonomic units [OTUs], and ASVs) across oil and Corexit treatments was also previously observed in both temperate (12) and Arctic (3) regions, further suggesting that some organisms may be capable of degrading both oil and Corexit. This also agrees with prior findings by McFarlin et al. (3) that the expression of some oil degradation genes (e.g., *alkB*) in Arctic seawater increased in response to exposure to oil or Corexit in incubations where they were provided individually.

The findings of this study demonstrate significant interactive effects during the biodegradation of crude oil and components of Corexit 9500 on chemical losses and microbial community structure in Arctic seawater. We found no evidence that Corexit suppressed crude oil biodegradation, although, conversely, the presence of oil negatively impacted DOSS degradation. Chemical and microbial community data showed that in the presence of both oil and Corexit, the nonionic surfactants (Span 80, Tween 80, and Tween 85) were degraded first, followed by labile oil component degradation. This apparent preferential degradation of dispersant components did not result in significant reductions in oil loss. This may be due to the reduction in oil droplet size from Corexit application outweighing the effects of competitive degradation or to the relatively small quantity of carbon contributed by Corexit compared to that of oil when applied in the 1:10 dispersant/oil ratio being insufficient to outcompete the utilization of petroleum hydrocarbons. Conclusions regarding the degradation of other Corexit components such as DOSS and DGBE remain unclear due to analytical variability and lack of data. The sequence of chemical degradation observed was associated with shifts in microbial community structure over time, where the oil+Corexit treatment was first dominated by taxa stimulated by Corexit only before shifting toward increased dominance by taxa stimulated by oil only. Some taxa (*Oleispira*, *Pseudofulvibacter*, and *Roseobacter*) responded to both oil and Corexit and shared the same ASVs across treatments, suggesting that some organisms are capable of utilizing components from both. Future research should focus on studying the longer-term fate of less-labile components of Corexit 9500 (DOSS, DGBE, and solvents) and the metabolic processes underlying oil and Corexit codegradation in seawater following chemical dispersion of oil to better understand the fate of dispersants and mechanisms of their biodegradation.

## MATERIALS AND METHODS

### Seawater collection and handling.

Arctic surface seawater collection was performed ∼1 km offshore of Utqiaġvik (formerly Barrow), AK, from the Chukchi Sea in August 2016. The 150 liters of collected seawater were stored at 5°C overnight and immediately transported by air to Fairbanks, AK, where it was aerated overnight at the temperature recorded at the time of collection (4°C) prior to the initiation of the incubation experiment. Before aliquoting seawater into mesocosms, it was supplemented with 16 ppm of Bushnell-Haas medium (2) to prevent potential nutrient limitations that may occur as an artifact of the small-scale incubations. The supplementation with Bushnell-Hass medium provided an additional measured 62 μM phosphate, 49 μM ammonia, 42 μM nitrate, and estimated 1.5 μM iron (see Table S1 in the supplemental material). Since these nutrient concentrations are much higher than those expected in Arctic regions (56, 57), the oil and Corexit component degradation reported here represents a best-case scenario.

### Incubation experiments.

Mesocosm incubation experiments were performed in a cold room set to a temperature of 4°C and the lights set to a 19-h day/5-h night cycle to mimic the conditions at the sampling site at the time of collection. Mesocosms were constructed by aliquoting 800 ml of seawater into acid-washed and preautoclaved 1-liter glass bottles containing Teflon-coated magnetic stir bars and were treated with either 50 ppm Alaska North Slope (ANS) crude oil, 5 ppm Corexit 9500 (1:10 dispersant-to-oil ratio), both, or neither (negative control). These oil concentrations and dispersant-to-oil ratio was chosen due to constraints of the detection limits of Corexit surfactant compounds to allow for quantitation of its degradation over time; however, both of these are within the range of expected values for dispersant application (58) and subsequent oil concentrations (59). Dispersant was applied with vigorous stirring, after which, the bottles were stirred at a low speed to allow movement of the oil slick at the surface but to prevent the formation of a large vortex, and the lids were left slightly ajar to allow air exchange. Sterile controls consisting of autoclaved seawater were used to account for abiotic losses of oil and Corexit from processes such as evaporation, volatilization, hydrolysis, and photooxidation. Treatments were replicated in triplicates and destructively harvested at 0, 5, 10, 20, and 30 days for crude oil and microbial analyses. No 5- or 20-day mesocosms for Corexit analyses were constructed because of space limitations; however, an additional triplicate series of larger 6-liter mesocosms were subsampled at high frequency to capture the relatively rapid degradation of the nonionic surfactant components of Corexit 9500 reported by McFarlin et al. (3). Treatments for the subsampled series included live and sterile abiotic treatments of seawater amended with 5 ppm Corexit with or without 50 ppm ANS crude oil. These large-sized incubations were subsampled through a Teflon tube with a syringe at 0, 1, 2, 3, 4, 5, 6, 7, 10, 20, and 30 days. In addition to acid washing and autoclaving, all vessels used for the analysis of Corexit were baked at 400°C for 12 h to remove any surfactant contaminants present as a result of manufacturing or other contamination sources.

### Chemical analysis of crude oil.

Chemical extraction and analysis of petroleum hydrocarbons using gas chromatography-mass spectrometry (GC-MS) were performed based on the methods of Prince and Douglas (60). Briefly, three 20-ml aliquots of dichloromethane were added to each mesocosm, mixed with a magnetic stir bar, pipetted off, and combined. The extracts were then dried with anhydrous sodium sulfate to remove any remaining water and stored at −20°C until analysis. Hydrocarbon analysis was performed on an HP 5890/5973 GC/MSD in scan mode with all signals normalized to the internal marker compound 17α(H),21β(H)-hopane, which is naturally present in oil and not biodegraded under normal environmental conditions (61, 62). Total petroleum hydrocarbons (TPH) were measured, as well as total *n*-alkanes, branched alkanes, and polycyclic aromatic hydrocarbons (PAHs), using the respective primary and secondary ions to identify those compounds (63).

### Chemical analysis of Corexit 9500.

Dispersant components were analyzed by liquid chromatography-tandem mass spectrometry (LC-MS/MS) in whole-bottle mesocosm and subsampled 6-liter incubations for the following compounds using the methods previously described by McFarlin et al. (3): DOSS, Tweens 80 and 85, Span 80, and ethylhexyl sulfosuccinate (EHSS). DOSS, Tweens 80 and 85, and Span 80 are known constituents of Corexit 9500 (18), and EHSS is a degradation metabolite of DOSS (4, 18). The Tweens could not be quantitated individually and were therefore quantitated as the sum of the concentrations of both (18). Percent recovery for this method ranged from 88% to 119%, and precision, reported as the relative standard deviation, ranged from 1.4% to 23%, depending on the analyte (18).

### Nutrient concentration analysis.

Mesocosms destined for microbial community analysis were also used to monitor nutrient concentrations. At each harvest time point, pH and dissolved oxygen were measured in each microcosm using a multimeter probe. Microcosms were then vacuum filtered on a 0.22-μm filter to separate cells from the bulk solution. The filters were immediately frozen at −80°C for microbial community analysis. The liquid filtrate was collected and frozen at −20°C for nutrient analysis. Nutrient analyses were performed for phosphate, silicate, nitrate, nitrite, and ammonium ions on a Seal Analytical AA3 at the University of Washington Marine Chemistry Laboratory according to the protocols of the WOCE Hydrographic Program (64).

### Microbial community analysis.

Microbial community analyses were performed in triplicates (*n* = 3) for each treatment and time point. DNA was extracted from frozen filters using a DNeasy PowerWater commercial extraction kit according to the manufacturer’s protocol (Qiagen, Venlo, Netherlands). To study the prokaryotic community structure, the V4 region of the 16S rRNA gene was amplified using indexed 515F (5′-GTGCCAGCMGCCGCGGTAA-3′) and 806RB (5′-GGACTACNVGGGTWTCTAAT-3′) primers (65) and sequenced on an Illumina MiSeq using a 2 × 250-bp format. Sequences were filtered, trimmed, dereplicated into 100% similarity amplicon sequence variants (ASVs), and assigned taxonomy from the SILVA rRNA database (v. 132) using the DADA2 bioinformatics pipeline (66–68).

### Statistical analyses.

Statistical significance for all chemical data between different treatments was determined with ANOVA and Tukey’s honestly significant difference tests using the JMP statistical software package (JMP, version 13.2.1; SAS Institute Inc., Cary, NC).

Microbial community data analyses were performed using the PC-ORD V6 statistical software package (PC-ORD v. 6.255 beta; MjM Software Design, Gleneden Beach, OR) (69). Nonmetric multidimensional scaling (NMDS) (70) plots were used to visualize differences in community structure between treatments and over time and statistical significances were determined using PERMANOVA (71) and nonparametric multiresponse permutation procedure (MRPP) tests (72). Community composition source was quantitated using the SourceTracker R statistical package (v. 1.0.1) (43), and unique ASVs were screened for and compared between treatments using an indicator species analysis (73). Correlations and modeling of chemical data to community structure were performed using Mantel tests and backward stepwise model selection (74).

### Data availability.

All 16S rRNA gene sequences were deposited in the National Institutes of Health Sequence Read Archive database under accession number PRJNA622244 (75).

## Supplementary Material

Supplemental file 1
