# Supplementary material for: The Interactive Effects of Crude Oil and Corexit 9500 on Their Biodegradation in Arctic Seawater
Source: Appl Environ Microbiol. 2020 Oct 15;86(21):e01194-20. doi: 10.1128/AEM.01194-20 (PMC7580538; doi:10.1128/AEM.01194-20)
Supplement: Supplemental file 1 [file AEM.01194-20-s0001.pdf]

Supplementary Information

Nutrient	Unamended	Amended
PO_4^{-3}	0.66	62.97
$Si(OH_4)$	12.53	10.73
NO_3^-	0.19	42.1
NO_2^-	0.03	0.04
NH_4^+	0.05	48.68
Chlorophyll a, uG/L	1.014	
Phaeopigments, uG/L	0.338	
Dissolved Organic Carbon	96.7	
Salinity, psu	28.81	
Total Suspended Solids, mg/L	5.68	
Total Phosphorus	2.06	
Total Nitrogen	14.06	
Turbidity, NTU	3.53	

SI Table 1. Nutrient concentrations of seawater after collection (unamended) and following the addition of 16 ppm Bushnell-Haas media (amended). All concentrations are in µM unless otherwise noted.

Treatment	Time (days)	*Total Petroleum Hydrocarbons* Total loss	*Total Petroleum Hydrocarbons* Abiotic loss	*n-alkanes* Total loss	*n-alkanes* Abiotic loss
Oil	5	12.1±0.9	8.3±1.3	23.9±3.1	14.7±4.1
	10	17.4±1.0	4.7±4.1	83.4±2.0	23.2±2.5
	20	22.8±4.9	10.7±4.3	87.6±4.7	29.9±3.7
	30	26.3±2.8	11.6±0.6	91.4±3.6	25.8±0.4
Oil+Corexit	5	9.0±3.9	8.0±4.4	20.4±2.9	15.1±4.3
	10	22.2±2.7	8.9±2.3	81.6±6.6	22.3±4.6
	20	27.5±5.4	13.9±0.3	90.7±2.2	27.9±2.3
	30	32.3±3.8	13.4±1.9	93.1±1.3	25.9±2.2

Treatment	Time (days)	*Branched alkanes* Total loss	*Branched alkanes* Abiotic loss	*Polycyclic Aromatic Hydrocarbons* Total loss	*Polycyclic Aromatic Hydrocarbons* Abiotic loss
Oil	5	16.6±1.7	6.0±5.0	20.3±1.6	12.6±2.3
	10	65.9±1.8	16.8±4.3	17.6±3.8	18.5±5.6
	20	72.5±6.0	24.6±5.9	34.0±3.1	30.3±4.9
	30	78.8±8.8	13.5±1.6	55.8±7.2	38.2±2.6
Oil+Corexit	5	12.8±1.9	5.7±7.1	12.2±3.8	12.4±3.5
	10	66.2±2.4	11.6±1.8	17.1±2.0	27.5±10.3
	20	76.5±1.8	17.1±2.3	39.9±9.5	38.2±5.3
	30	79.7±4.7	14.5±4.1	54.1±2.8	38.0±2.0

SI Table 2. Mean and 1 standard deviation of total (biotic+abiotic) and abiotic percent losses of crude oil compounds.

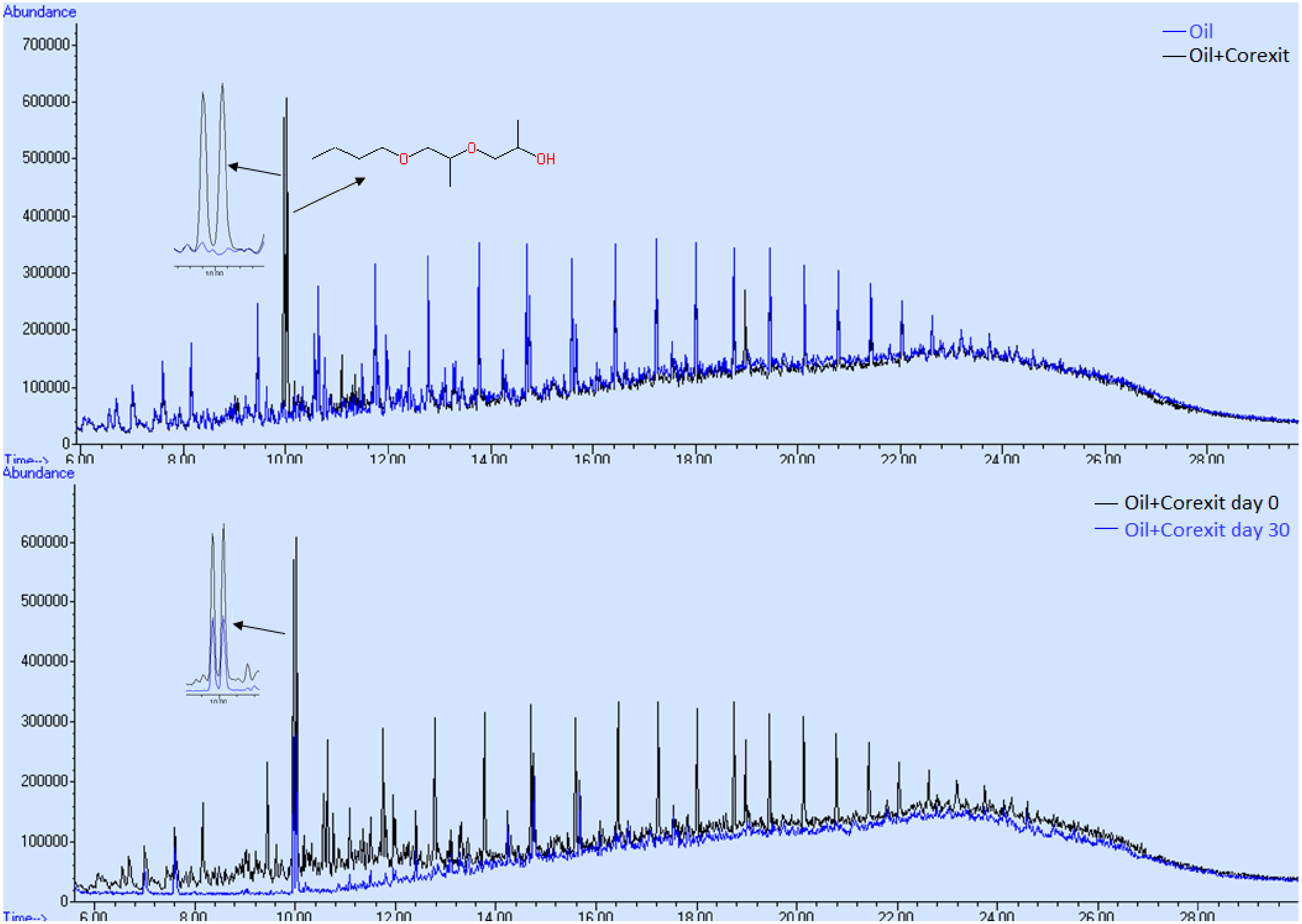

SI Figure 1. GC/MS chromatogram of oil with Corexit samples a) showing an unknown peak identified as dipropylene glycol n-butyl ether (DGBE) that was not present in oil-only samples and b) which remained present in significant amounts by the end of the 30 day incubation.

	Pre-processing	Post-processing
Total sequences	3,907,863	3,513,411
Min sequences per sample	43,899	41,649
Max sequences per sample	135309	116,679
Average sequences per sample	76,625	67,566
Number of ASVs	7,541	4,042

SI Table 3. Summary of 16S rRNA gene sequences grouped at 100% sequence similarity (Amplicon Sequence Variants, ASVs) output from the dada2 pipeline (pre-processing) and after removal of chloroplast and singleton ASVs (post-processing).

	Genus	Corexit	Oil	Oil+Corexit	Control	p value
ASV0002	*Oleispira*	21	24	45	9	0.0006
ASV0003	*Oleispira*	22	24	48	5	0.002
ASV0004	*Oleispira*	27	28	39	5	0.0384
ASV0008	*Oleispira*	22	24	45	10	0.0008
ASV0010	*Oleispira*	26	27	40	6	0.0282
ASV0011	*Oleispira*	22	24	48	5	0.0016
ASV0012	*Oleispira*	21	26	44	7	0.0004
ASV0018	*Oleispira*	25	26	43	4	0.0036
ASV0020	*Oleispira*	21	26	44	8	0.0004
ASV0025	*Oleispira*	24	26	44	4	0.0018

SI Table 4. Indicator values for the 10 most abundant ASVs identified as an indicator species ($p<0.05$) when comparing all treatments in a blocked indicator species analysis factoring in the effect of time.

	Mantel correlation coefficient (r)	p value
	All samples	
**Time**	**0.597**	**0.0010**
$Si(OH)_4$	0.182	0.0070
NO_2	0.268	0.0010
NH_4	0.236	0.0020
pH	0.347	0.0010
DO	0.101	0.0200
	Day 5	
No significant ($p<0.05$) relationships observed		
	Day 10	
NH_4	0.372	0.0030
pH	0.391	0.0090
DO	0.374	0.0170
	Day 20	
NO_2	0.345	0.0460
	Day 30	
$Si(OH)_4$	0.289	0.0330
NO_3	0.233	0.0400
**DO**	**0.619**	**0.0010**

	Mantel correlation coefficient (r)	p value
	Oil-containing treatments	
**Time**	**0.774**	**0.0010**
**TPH**	**0.758**	**0.0010**
**n-Alkanes**	**0.681**	**0.0010**
**Branched alkanes**	**0.728**	**0.0010**
**PAHs**	**0.669**	**0.0010**
PO_4	0.253	0.0250
$Si(OH)_4$	0.281	0.0071
NO_3	0.315	0.0050
NO_2	0.396	0.0010
**pH**	**0.620**	**0.0010**
	Corexit-containing treatments	
**Time**	**0.588**	**0.0010**
EHSS	-0.164	0.0160
DOSS	0.273	0.0320
NO_3	0.416	0.0010
NO_2	0.355	0.0330
pH	0.384	0.0020
DO	0.205	0.0470

SI Table 5. Significant relationships of environmental parameters to the prokaryotic microbial community structure determined using Mantel tests. Moderate to strong correlations ($r>0.5$) are in bold. Note that the following parameters are strongly correlated with time: TPH ($r=0.775$, $p<0.0001$), n-alkanes ($r=0.573$, $p<0.0001$), branched alkanes ($r=0.638$, $p<0.0001$), PAHs ($r=0.978$, $p<0.0001$), and DOSS ($r=0.801$, $p<0.0001$).

Final model	R^2	Adjusted R^2	p values
Amylibacter=DOSS+Time+(DOSS*Time)	0.65	0.59	Overall<0.0001<br>DOSS=0.0007<br>Time=0.0433<br>DOSS*Time=0.0189
Colwellia=PAHs	0.30	0.27	Overall=0.0051
Moritella=Treatment	0.71	0.69	Overall<0.0001
Octadecabacter=DOSS+(DOSS*PAHs)	0.88	0.85	Overall<0.0001<br>DOSS=0.0002<br>DOSS*PAHs<0.0001
Oleispira=n-alkanes+DOSS	0.95	0.94	Overall<0.0001<br>n-alkanes=0.0009<br>DOSS=0.0118
Polaribacter=DOSS+(DOSS*pH)	0.80	0.78	Overall<0.0001<br>DOSS<0.0001<br>DOSS*pH<0.0001
Pseudofulvibacter=EHSS+NO_3+PO_4	0.71	0.66	Overall<0.0001<br>EHSS<0.0001<br>NO_3=0.0024<br>PO_4=0.0427
Roseobacter=PAHs+DOSS+NH_4	0.93	0.90	Overall<0.0001<br>PAHs<0.0001<br>DOSS<0.0001<br>NH_4=0.0051
Sedimentalea=PAHs+EHSS+(PAHs*EHSS)	0.90	0.86	Overall=0.0002<br>PAHs<0.0001<br>EHSS=0.0024<br>PAHs*EHSS=0.0081
Thalassolituus=TPH+NO_3+PO_4	0.90	0.88	Overall<0.0001<br>TPH=0.0003<br>NO_3<0.0001<br>PO_4=0.0003

SI Table 6. Backward stepwise final models selected for individual genera using the lowest corrected Akaike and Bayesian Information Criterion (AICc and BIC).

	Genus	Corexit	Oil	Control	p value
ASV 49	*Oleispira*	11	70	4	0.001
ASV 74	*Oleispira*	10	70	4	0.0006
ASV 82	*Oleispira*	12	70	4	0.0008
ASV 86	*Moritella*	85	0	3	0.0002
ASV 98	*Moritella*	86	0	1	0.0002
ASV 101	*Moritella*	97	0	0	0.0002
ASV 148	*Moritella*	89	0	0	0.0002
ASV 149	*Moritella*	96	1	0	0.0002
ASV 156	*Moritella*	88	0	0	0.0002
ASV 161	*Moritella*	86	2	0	0.0002
ASV 165	*Moritella*	89	0	0	0.0002
ASV 188	Unclassified Spongiibacteraceae	83	0	0	0.0002
ASV 206	*Moritella*	100	0	0	0.0002
ASV 228	*Moritella*	89	0	0	0.0002
ASV 239	*Moritella*	91	0	0	0.0002
ASV 287	*Moritella*	100	0	0	0.0002
ASV 631	*Colwellia*	74	4	1	0.0002
ASV 813	Unclassified Colwelliaceae	81	1	1	0.0002

SI Table 7. Summary of results of a blocked indicator species analysis factoring in the effect of time for ASVs with $p<0.05$ and indicator values (IV)>70 for oil-only and Corexit-only treatments compared to the control.

	Genus	Corexit	Oil	Oil+Corexit	Control	p value
ASV0049	*Oleispira*	6	39	43	2	0.0226
ASV0074	*Oleispira*	6	39	44	2	0.0234
ASV0082	*Oleispira*	6	39	44	2	0.0164

SI Table 8. Indicator values from a blocked indicator species analysis of all treatments for ASVs previously identified as indicator species for oil-only treatments.

	Genus	Corexit	Oil	Oil+Corexit	Control	p value
ASV0086	*Moritella*	48	0	40	2	0.0012
ASV0098	*Moritella*	52	0	37	1	0.0006
ASV0101	*Moritella*	66	0	27	0	0.0002
ASV0148	*Moritella*	52	0	35	0	0.0004
ASV0149	*Moritella*	66	1	26	0	0.0002
ASV0156	*Moritella*	54	0	36	0	0.0002
ASV0161	*Moritella*	52	1	36	0	0.0002
ASV0165	*Moritella*	62	0	25	0	0.0002
ASV0188	Unclassified Spongiibacteraceae	79	0	2	0	0.0002
ASV0206	*Moritella*	62	0	32	0	0.0002
ASV0228	*Moritella*	62	0	25	0	0.0002
ASV0239	*Moritella*	53	0	38	0	0.0002
ASV0287	*Moritella*	63	0	31	0	0.0002
ASV0631	*Colwellia*	52	3	25	1	0.0002
ASV0813	Unclassified Colwelliaceae	51	1	31	1	0.0004

SI Table 9. Indicator values from a blocked indicator species analysis of all treatments for ASVs previously identified as indicator species for Corexit-only treatments.
